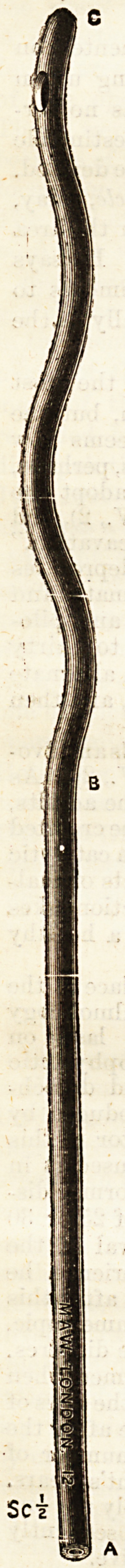# New Appliances and Things Medical

**Published:** 1896-11-14

**Authors:** 


### NEW APPLIANCES AND THINGS MEDICAL.

[We shall be glad to receive, at our Office, 28 & 29, Southampton Street, Strand, London, W.C., from the manufacturers, specimens of all new preparations and appliances which may be brought out from time to time.]

### NEW CATHETER.

(Messrs. Maw, Son, and Thompson.)

C

B

Sc $\frac{1}{2}$ A

The accompanying illustration shows a new form of catheter made at the suggestion of Mr. Tom Smith by Messrs. Maw, Son, and Thompson. The body of the catheter is of fine soft rubber with a solid end, but the upper half is stiffened with a quill to facilitate the manipulation during introduction and to enable it to be held with greater ease. The advantages of a really soft catheter of this nature over what are usually called soft catheters, but which are nearly as irritating to the mucous membrane as the silver variety, are as obvious to the patient as to the surgeon. This new form of catheter appears already to have many advocates, and in a short time will probably become the general possession of practitioners.

### CHLOROS. AN ANTISEPTIC AND DEODORIZER.

(United Alkali Co., Limited, Exchange Buildings, London).

Among the host of new antiseptics being daily put before the public, one is apt to forget the old and well-tried varieties. For instance, Chloros, which is really hypochlorite of soda with 10 per cent. available chlorine, is one of the oldest deodorizers known to science. Before the meaning of antiseptics was known hypochlorite of soda was largely used for domestic purposes for cleaning closets, drains, &c. Its germicide powers have more recently been accurately gauged and found to be most effectual in the case of pathogenic as well as other varieties of bacteria, and that, too, in very dilute solution it may be safely relied upon for killing many germs commonly met with. In addition, its oxidising or deodorizing powers are peculiarly useful in the case of offensive odours arising from defective drains, cesspools, and other sources. The advantages of Chloros are that it is not an active poison, it leaves no insoluble residue, and it does not stop up drains or stain porcelain or earthenware.

### TRITIKOLA BISCUITS, TRITICUMINA BREAD AND FOOD, &c.

(Meaby and Co., Limited, Reading.)

We have received samples of the above from Messrs. Meaby and Co., and after trial and examination of the preparations we are enabled to report most favourably on all the specimens which have been submitted to us. The Triticumina bread is now so well known to the public and to the profession that we can add little that is not already familiar. It is a brown bread of fine quality, containing all the constituents of the entire grain, and consequently a higher percentage of phosphates than is present in ordinary wheaten bread. The percentage of soluble carbohydrates also compares very favourably with that of ordinary bread. For invalids and those of weak digestion Triticumina bread has the following advantages; It is highly nutritious, can be easily digested and absorbed, and is free from coarse particles of bran and other forms of indigestible cellulose; and further, after a daily consumption during some years, we still hold none other so palatable. With regard to the Triticumina food, being a malted food of excellent quality, it is suited for young children and invalids, who are unable to digest ordinary farinacious foods, consisting of unaltered starch; in addition it contains a high percentage of phosphates and albuminous substances. The Tritikola biscuits are quite a new departure in preparations of this kind, since they possess sustaining qualities which make them of peculiar value as a concentrated food and tonic for those undergoing heavy exertion on the march, or on other occasions. They have been particularly recommended for cyclists undertaking long rides, and being capable of being packed into a small space or carried in the pocket, they appear to us to be well adapted for this purpose. It is claimed for them that they are prepared from malt, albumen, farina, caffeine, saccharine, and extract of kola nut, and we have proved to our satisfaction that their complex composition is a most happy combination of nutritive and tonic principles, and such as to be highly appreciated by those enduring great muscular fatigue.

### URANIUM NITRATE PALATINOIDS.

(Messrs. Oppenheimer and Co., Limited, 14, Worship Street, E.C.)

We have received samples of the above preparation. Salts of uranium being particularly nauseating must, when prescribed, be administered in some carefully-disguised form. The palatinoids of the nitrate contain a dose of two and a-half grains, Uranium nitrate has lately acquired a considerable reputation as a drug which exercises some control over the excretion of sugar in Diabetes Mellitus, and Dr. West's recent paper on its applicability in this intractable disease will probably induce many practitioners to give it a trial. Messrs. Oppenheimer's elegant preparation is, therefore, well timed, and will probably be in extensive demand.

### A SLING FOR CARRYING COMPRESSED GASES.

Mr. Abbot, of Aberford, Yorkshire, has devised a sling for carrying a cylinder of compressed oxygen, for the use of rescue parties in case of colliery explosions. His personal experience in going down with the rescue parties after the frightful colliery explosion at Michlefield in May of this year not only convinced him of the great restorative value of ogygen in cases of poisoning by after-damp, which is now known to owe much of its evil effects to carbonic oxide, but also of the practical impossibility of dragging large cylinders over the wreckage so often encountered under such circumstances. Small cylinders are required, provided with the necessary face piece, and so slung as to leave the arms of the operator free. These requirements are well met by the sling contrived by Mr. Abbott, which is made by Messrs. Reynolds and Branson, of Leeds.

## Figures and Tables

**Figure f1:**